# Transition of milk fatty acid profile and vitamins A and E from colostrum to mature milk in Danish Holstein cows

**DOI:** 10.1371/journal.pone.0328897

**Published:** 2025-08-14

**Authors:** Saman Lashkari, Lauryn Charoy, Laurie Pons, Søren K. Jensen

**Affiliations:** 1 Department of Animal and Veterinary Sciences, Aarhus University, AU Viborg-Research Centre Foulum, Tjele, Denmark; 2 Department of Animal Sciences and Livestock Systems, School of Engineering, Toulouse, France; Alexandria University, EGYPT

## Abstract

Colostrum, the initial milk produced by mammals after giving birth, has evolved to serve as a primary rich source of essential nutrients for newborns. However, there are considerable changes in nutrient composition from colostrum to mature milk. Colostrum and transition milk have specific fat-soluble vitamin levels, fat level, and fatty acid (FA) composition compared to mature milk, and this unique fatty acid composition of colostrum may reflect the physiological needs of newborn calves. The aim of this study was to investigate changes in the FA profile and levels of vitamins A and E from colostrum to mature milk. Colostrum or milk samples from the 1^st^ (colostrum), 2^nd^, 3^rd^, 4^th^, 5^th^, 6^th^, and 13^th^ (as a sample of mature milk) milking were collected from 10 multiparous Danish Holstein cows. The level of vitamin A was significantly affected by milking time (*p* < 0.001), with the highest level in the colostrum (2.3) and the lowest in mature milk (0.5 µg/g). Vitamin E level was significantly affected by milking time (*p* < 0.001), with the highest level in the colostrum (14.8) and the lowest in mature milk (1.8 µg/g). The proportion of C16:0 FA decreased from colostrum to mature milk while the proportion of C18:0 FA increased from the colostrum to mature milk. The proportion of n-6 FA (C18:2n-6, C18:3n-6, C20:3n-6, C20:4n-6, and C22:5n-6) decreased from colostrum to mature milk (*p* < 0.001 for all FA). However, the proportion of C18:3n-3 FA increased from colostrum (3.0) to mature milk (5.9 g/kg of FA; *p* < 0.001), while proportions of C20:5n-3, C22:5n-3, and C22:6n-3 FA decreased from colostrum to mature milk (*p* < 0.001 for all FA). The proportion of trans 18:1n-10 (*p* = 0.005) and trans 18:1n-11 (*p* < 0.001) was affected by milking time and increased from colostrum to mature milk. In conclusion, there is a considerable higher vitamin A and E levels in colostrum compared to mature milk, and vitamin A and E levels were reduced from colostrum to mature milk. Additionally, there were significant changes in the FA profile from colostrum to mature milk, with an increase in C18:3n-3 FA and a decrease in C18:2n-6 FA.

## Introduction

Colostrum, the initial milk produced by mammals after giving birth, possesses a unique composition that significantly differs from mature milk. Colostrum has evolved to serve as the primary source of essential nutrients for newborns. Colostrum contains 50% more fat and nearly five times the protein content compared to mature cow’s milk [1]. The fat content provides crucial energy for thermoregulation, while the protein primarily consists of immunoglobulins. These immunoglobulins offer passive immunity by providing antibodies against pathogens. Similar to fat and protein, vitamin A level [2] is higher in colostrum than in mature milk (75 in colostrum vs 39 µg/ml mature milk). Vitamin E [3] is also higher in colostrum than mature milk (98 in colostrum vs to 20 µg/g of fat in mature milk). A limited placental transfer of fat-soluble vitamins during gestation justifies the high levels of vitamins A and E in colostrum and highlights the importance of colostrum feeding for the mammalian infant [4].

According to Zarcula et al. [1], the composition of bovine colostrum can be influenced by various factors, including the cow’s breed, number of lactation, diet, duration of the dry period, and days in milk (DIM). The biological role of colostrum and its composition shift during the days following parturition as it transforms into mature milk [5]. The definition of time periods associated with each of these stages varies considerably in the literature, from classification as colostrum immediately after parturition, to also include between 5 and 7 days post-partum [6]. O’Callaghan et al. [6] demonstrated a distinct change in FA profile from colostrum to mature milk, reporting a significantly higher proportion of C18:0 in mature milk compared to colostrum at the expense of C16:0. In addition, proportions of C18:2n-6 and C18:3n-3 in total FA reduced from colostrum to milk [6]. However, knowledge on changes in proportions of long chain unsaturated fatty acids and fat-soluble vitamins from colostrum to milk is limited. The objective of this study was to investigate the transition in the FA profile, vitamin A, and vitamin E from colostrum to mature milk in Danish Holstein cows.

## Materials and methods

### Ethics approval

The present study was carried under the animal experimentation permit number 2020-15-0201-00709 granted by The Animal Experiments Inspectorate of the Ministry of Food, Agriculture and Fisheries of Denmark, in accordance with Justice Law No 474 (November 2014) concerning animal experiments and the care of experimental animals. This legislation aligns with EU standards for the protection of animals used for scientific purposes.

### Animals and experimental design

Ten multiparous Danish Holstein cows from the dairy cattle research farm at Aarhus University in AU-Viborg, Foulum were fed the same diet during dry-off, close-up, and early lactation (Table 1). Length of close-up period was 20.8 ± 5.1 days (means ± standard deviation). Three days before expected calving date, cows were moved to an individual calving pen with straw bedding, and after calving, cows and calves were kept in the same calving pen for 12–24 h. Six consecutive colostrum or transition milk samples were collected from both morning and evening milking on 1 (1^st^ and 2^nd^ milking), 2 (3^rd^ and 4^th^ milking), and 3 (5^th^ and 6^th^ milking) DIM, and a mature milk sample was collected from the evening milking on 7 DIM (either 13^th^ or 14^th^ milking), which presented as 13^th^ milking in table and figures. The initial colostrum sample (1^st^) was taken after calving in the calving pen, while the remaining colostrum and milk samples were collected in milking parlor with 12 hour interval. All milk and colostrum samples were stored at −18°C for subsequent analysis. Cows were housed in a loose-housing system with deep straw bedding during close-up and concrete floor and cubicles during lactating periods. All cows were provided with ad libitum access to feed (7–10% orts), and the daily meals were divided into two meals fed at 0800 and 1600 hours. The cows were provided unrestricted access to water. Daily feed intake was recorded throughout the entire experiment by using Insentec feed troughs. Weekly sample of total mixed ration was taken to measure the DM content (60°C for 48h), and daily dry matter intake (DMI) was calculated by multiplying feed intake by DM content.

**Table 1:** Total mixed ration and nutrient composition of diets.

	Diets		
	Far-off	Close-up	Lactating
**Feed ingredients, g/kg DM diet**			
Grass silage	912	–	179.7
Corn silage	–	741.1	344.2
Spring barley straw	80.0	–	–
Barley straw	–	–	153.0
Corn, finely ground	–	74.9	–
Rapeseed Cake, 10.5% fat	–	104.8	130.0
Rapeseed meal, 4% fat	–	–	84.1
Corn gluten	–	59.9	–
Sugar beet pellets	–	–	91.8
Salt	–	–	1.8
Sodium bicarbonate	–	–	9.9
Chalk	–	1.9	0.7
Magnesium chloride hexahydrate	–	4.8	–
Calcium chloride dihydrate	–	5.2	–
Mineral and vitamin premixed	8.0	7.5	3.7
Vitamin A, E, and D supplements	–	–	1.1
**Calculated nutrient composition**			
Energy, MJ/kg DM	5.29	7.31	6.56
Crude protein, g/kg DM	119	149.9	163
Fatty acids, g/kg DM	16	27	26
NDF, g/kg DM	467	310	320
Starch, g/kg DM	0	262	193
Total calcium, g/kg DM	7.9	4.5	4.10
Total phosphorus, g/kg DM	3.4	2.8	4.3
Total magnesium, g/kg DM	3.0	2.3	1.90
Dietary cation-anion balance, mEq/kg DM	294	−40	202

### Crude fat and FA analysis

Crude fat and FA from the colostrum and milk were extracted using a mixture of methanol and chloroform, following the method originally reported by Bligh et al. [7] and subsequently modified by Jensen [8]. To quantify the milk FA, C17:0 (nonadecanoic acid, Sigma-Aldrich) was used as internal standard. Our previous study showed that C17:0 had no effect on the FA composition or the total FA content [9]. The extracted FA were converted into FA methyl ester by adding 0.8 mL of NaOH (2%) in methanol, followed by addition of 1.0 mL of boron trifluoride reagent [10]. Finally, FA methyl ester was extracted with 2.0 mL heptane. Quantification of FA methyl ester was performed using a gas chromatograph (Hewlett Packard 6890, Agilent Technologies) equipped with a capillary column of 60 m × 0.32 mm i.d., 0.25 μm thickness (Omegawax 320; Supelco, Sigma-Aldrich), with an automatic column injector (Hewlett Packard 7673), and a flame-ionization detector was used for quantifying the FA. The starting temperature was 170°C, gradually increased at a rate of 2°C per minute to reach 200°C, held steady for 5 minutes, and eventually elevated to 220°C at a rate of 5°C per minute. Peaks were identified by comparison of retention times with external standards (GLC 68C, Nu-Prep Chek) for long-chain FA.

### Vitamin A and E analysis

α-Tocopherol concentration of colostrum and milk samples was analyzed by high performance liquid chromatography (HPLC) after saponification and extraction into heptane [11]. The samples (1.00 mL) were diluted with 2.00 mL ethanol (96% v/v), 0.50 mL methanol (100%), 1.00 mL ascorbic acid (20% w/v), 0.30 mL KOH-water (1:1, w/v), and 0.70 mL water. Samples were saponified at 80ºC for 20 min and cooled in the dark. Tocopherol was extracted into two volumes of 5.00 mL heptane, and 100 μL of the combined heptane phase was injected into the HPLC. All solvents used were of HPLC quality. A Perkin-Elmer HS-5-Silica column (4.0 x 125 mm; Perkin-Elmer GmbH, D-7770 Überlingen, Germany) was used for tocopherol determination. The mobile phase was heptane containing 2-propanol (3.0 mL/L) and degassed with helium. The flow rate was 3.0 mL/min. The obtained peak areas and retention time were compared with Merck (D-6100 Darmstadt, Germany) external standards for identification and quantification of the tocopherol. Fluorescence detection was performed with an excitation wavelength of 290 nm and an emission wavelength of 327 nm.

Retinol in milk was determined as described by Jensen [12]. Briefly, 500 µL colostrum and milk was extracted and saponified by adding 1.0 mL ascorbic acid solution (20% w/v), 0.5 mL methanol, 2.0 mL ethanol, 1.0 mL KOH-water (1:1, w/v), and water to a final volume of 5.5 mL. The tubes were flushed with nitrogen, sealed, and their contents saponified by placement in a water bath at 70°C for 20 minutes, in the dark. Then the tubes were rapidly cooled in cold water, and 5 mL heptane-di-isopropyl ether (3:1) was added to each tube, and retinol was extracted into the heptane-di-isopropyl ether layer by shaking the tubes on a vibrator (IKA-Vibrax-VXR, Janke & Kunkel, Germany) for one min. After centrifugation for 5 min at 1000 g the heptane-di-isopropyl ether layer was carefully transferred into a new culture tube. The extraction procedure was repeated with another 5 mL portion of heptane-di-isopropyl ether (3:1) and 100 μL of the combined extract was injected into the HPLC. The HPLC column consisted of a 4.0 x 125 mm Perkin Elmer HS-5-Silica column, and the mobile phase was heptane modified with 2-propanol (6%). The mobile phase was degassed by means of helium prior to use. The flow rate was 3.0 mL per min, and the column temperature was held at room temperature. Fluorescence detection was performed with an excitation wavelength of 344 nm and an emission wavelength of 472 nm.

### Statistical analysis

The obtained data (milk yield, vitamins A and E, crude fat, total FA, and individual FA proportion) were analyzed by using proc mixed procedure in SAS (version 9.4; SAS Institute) with Maximum Likelihood method, and the model was:


$$Y_{ij}=\;\mu \;+\;M_{i}\;+\;c_{j}\;+\;e_{ij}$$

where Y was the dependent variable, *µ* was the overall mean, *M* was fixed effect of milking time (i: 1^st^, 2^nd^, 3^rd^, 4^th^, 5^th^, 6^th^, and 13^th^), and *c* was the random effect of cow (j; 10 cows as experimental unit), and the *e*_ij_ was random error. The same model was used for statistical analysis of DMI during the first week post-partum, and the difference was that, instead of milking time in the previous model for milk yield composition, the DIM (0, 1, 2, 3, 4, 5, 6, and 7 DIM) was included in the model.

For DMI during close-up and lactation phase, weekly average of DMI was used in the statistical analysis (three weeks of close-up and the first week of lactation). The DMI was analyzed by using proc mixed procedure in SAS with Maximum Likelihood method, and the model was:


$$Y_{ij}\;=\;\mu \;+\;{FF}_{i}+\;c_{j}\;+\;e_{ij}$$

where Y was the dependent variable, *µ* was the overall mean, *FF* the fixed effect of feeding phase (i: close-up and lactating phases) and c was random effect of cow (j; 10 cows as experimental unit), and the *e*_ij_ was random error. The approximation for degree of freedoms was specified by DDFM = SATTERTHWAITE option in the stated model. Except for Table 1, values presented in Table 2 and figures are LS-means with corresponding SEM. All the collected data were included in the data analysis. Significance was declared at *p* ≤ 0.05 and a tendency at 0.05 < *p* ≤ 0.10. LS-means was compared by using adjusted Tukey test.

**Table 2:** Changes in crude fat, total FA, and FA profile from colostrum (1^st^ milking) to mature milk (13^th^ milking; n = 10).

	Milking time							SEM^1^	*p*-values
	1^st^	2^nd^	3^rd^	4^th^	5^th^	6^th^	13^th^		
Crude fat, %	5.53	3.95	3.91	4.75	6.51	5.88	5.50	0.03	0.031
Total fatty acids, %	4.63	3.28	3.63	4.06	4.47	4.72	4.33	0.49	0.103
FA profile, g/kg of FA									
C4:0	14.52^b^	21.60^a^	19.30^ab^	21.08^a^	23.59^a^	24.68^a^	24.71^a^	1.56	< 0.001
C6:0	0.04^c^	0.13^abc^	0.09^bc^	0.17^ab^	0.13^abc^	0.16^ab^	0.20^a^	0.03	< 0.001
C8:0	6.36^b^	9.05^a^	8.66^a^	9.29^a^	9.89^a^	10.31^a^	10.16^a^	0.70	< 0.001
C10:0	16.22^b^	21.1^6ab^	20.99^ab^	21.94^a^	22.15^a^	21.94^a^	20.22^ab^	1.96	0.010
C11:0	0.22	0.28	0.25	0.30	0.31	0.32	0.29	0.04	0.354
C12:0	30.74^a^	30.35^a^	29.81^ab^	28.14^ab^	26.77^abc^	25.45^bc^	22.18^c^	2.29	< 0.001
C13:0	0.61	0.62	0.50	0.58	0.60	0.61	0.57	0.07	0.575
C14:0	155^a^	130^b^	127^b^	113^c^	104 cd	97^de^	83^e^	7	< 0.001
C14:1n-5	15.32^a^	11.01^b^	10.56^bc^	7.94 cd	6.87^d^	5.91^d^	5.55^d^	0.89	< 0.001
C15:0	7.40	7.62	7.60	7.79	7.62	7.38	7.20	0.45	0.453
C16:0	422^a^	373^b^	374^b^	338^c^	311^cd^	290^d^	260^e^	12	< 0.001
C16:1n-9	2.37	2.28	2.33	2.29	2.24	2.21	2.23	0.13	0.828
C16:1n-7	30.28^a^	27.21^ab^	27.02^ab^	25.48^ab^	24.47^b^	23.42^b^	23.97^b^	2.03	0.003
C17:1n-9	0.12	1.01	0.27	0.55	0.61	0.91	1.21	0.34	0.156
C18:0	64^e^	92^d^	90^d^	111^c^	126^bc^	143^ab^	157^a^	5	< 0.001
C18:1n-9	175^e^	214^d^	222^d^	254^c^	272^bc^	286^b^	319^a^	18	< 0.001
C18:1n-7	7.75^d^	9.50 cd	10.03^c^	11.96^b^	13.05^b^	13.55^ab^	14.96^a^	0.72	< 0.001
Trans C18:1n-11	0.79^d^	1.13^c^	1.21^bc^	1.39^ab^	1.49^a^	1.43^a^	1.60^a^	0.16	< 0.001
Trans C18:1n-10	0.12^c^	0.17^bc^	0.19^bc^	0.26^abc^	0.30^abc^	0.39^ab^	0.44^a^	0.06	< 0.001
C18:2n-6	31.92^a^	29.13^b^	28.54^bc^	27.11^bc^	26.93^bc^	26.29^c^	26.05^c^	1.03	< 0.001
C18:3n-6	0.38^a^	0.31^ab^	0.27^bc^	0.23^bc^	0.18^c^	0.21^bc^	0.19^c^	0.03	< 0.001
C18:3n-3	3.03^e^	3.42^ed^	3.43^ed^	3.97 cd	4.41^bc^	4.82^b^	5.87^a^	0.29	< 0.001
Cis-9, trans-10 conjugated linoleic acid	2.50	2.61	2.84	2.77	2.81	2.87	2.92	0.23	0.616
C19:0	1.35	1.83	1.47	1.43	1.78	1.69	1.91	0.16	0.019
C20:0	0.62^c^	0.69^c^	0.76^bc^	0.90^bc^	1.09^ab^	1.32^a^	1.35^a^	0.13	< 0.001
C20:1n-9	0.45^c^	0.47^c^	0.61^bc^	0.72^ab^	0.78^ab^	0.77^ab^	0.79^a^	0.08	< 0.001
C20:2n-6	0.28	0.27	0.23	0.21	0.30	0.29	0.32	0.03	0.143
C20:3n-6	2.53^a^	2.00^b^	1.93^b^	1.49^c^	1.30^c^	1.13^c^	0.74^d^	0.14	<0.001
C20:4n-6	2.86^a^	2.78^a^	2.89^a^	2.67^ab^	2.42^ab^	2.18^bc^	1.69^c^	0.17	<0.001
C20:3n-3	0.05^ab^	0.01^b^	0.03^ab^	0.06^ab^	0.05^ab^	0.08^a^	0.08^ab^	0.02	0.044
C20:5n-3	1.25^a^	1.09^abc^	1.13^ab^	1.04^abc^	0.90^bc^	0.84^c^	0.58^d^	0.08	< 0.001
C22:0	0.26^abc^	0.23^bc^	0.19^c^	0.23^bc^	0.25^abc^	0.34^ab^	0.38^a^	0.03	< 0.001
C22:1n-9	0.70^a^	0.52^bc^	0.55^ab^	0.40^bcd^	0.37^cd^	0.33^d^	0.31^d^	0.05	< 0.001
C22:5n-6	0.36^a^	0.21^bcd^	0.29^ab^	0.25^bc^	0.21^bcd^	0.19^cd^	0.13^d^	0.03	< 0.001
C22:5n-3	2.68^a^	2.18^b^	2.12^b^	1.78^bc^	1.61^c^	1.45^c^	0.95^d^	0.13	< 0.001
C22:6n-3	0.30^a^	0.17^bc^	0.22^ab^	0.18^b^	0.17^bc^	0.16^bc^	0.05^c^	0.03	< 0.001
C24:0	0.15^ab^	0.12^ab^	0.09^b^	0.12^ab^	0.13^ab^	0.18^a^	0.19^a^	0.02	0.001
C24:1n-9	0.04	0.01	0.01	0.01	0.02	0.02	0.02	0.01	0.286
SCFA^2^	53.58	60.97	59.80	59.85	59.26	58.18	53.04	4.87	0.234
MCFA^3^	633^a^	553^b^	550^b^	496^c^	458^cd^	428^de^	384^e^	18	< 0.001
LCFA^4^	299^e^	364^d^	371^d^	423^c^	459^bc^	489^b^	538^a^	21	< 0.001
SFA^5^	704^a^	667^b^	662^bc^	633^cd^	613^de^	600^e^	565^f^	19	< 0.001
USFA^6^	296^f^	333^e^	338^de^	367^cd^	387^bc^	400^b^	435^a^	19	< 0.001
PUFA^7^	48.15^a^	44.19^b^	43.92^bc^	41.75^bcd^	41.28^bcd^	40.52^cd^	39.57^d^	1.37	< 0.001
n-9	179^e^	217d	225^d^	257^c^	276^bc^	289^b^	323^a^	18	< 0.001
n-7	38.03	36.70	37.05	37.44	37.52	36.97	38.93	2.30	0.901
n-6	38.34^a^	34.71^b^	34.15^bc^	31.96^bcd^	31.34^cd^	30.30^d^	29.12^d^	1.16	< 0.001
n-3	7.31	6.87	6.93	7.02	7.13	7.35	7.53	0.39	0.405
Ratio n-6/n-3	5.54^a^	5.19^ab^	5.03^abc^	4.61^bcd^	4.45^cde^	4.14^de^	3.89^e^	0.26	< 0.001

^1^SEM for milking time.

^2^SCFA = short chain fatty acids (C6 to C12).

^3^MCFA = medium chain fatty acids (C14 to C17).

^4^LCFA = long chain fatty acids (≥ C18).

^5^SFA = saturated fatty acids.

^6^USFA = unsaturated fatty acids.

^7^PUFA = polyunsaturated fatty acids.

a–f LS-means within a row with different superscripts differ according to the adjusted Tukey’s test (*p* < 0.05).

## Results

### DMI and colostrum and milk yield

**Fig 1:**
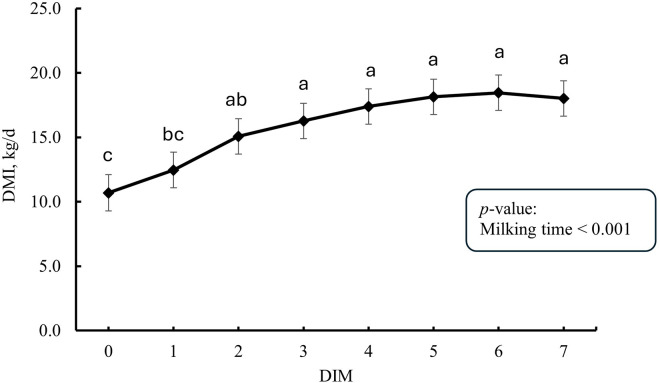
Daily DMI in the first week post-partum (n = 10). Error bars present SEM. ^a-c^ LS-means with different letters differ according to the adjusted Tukey’s test.

**Fig 2:**
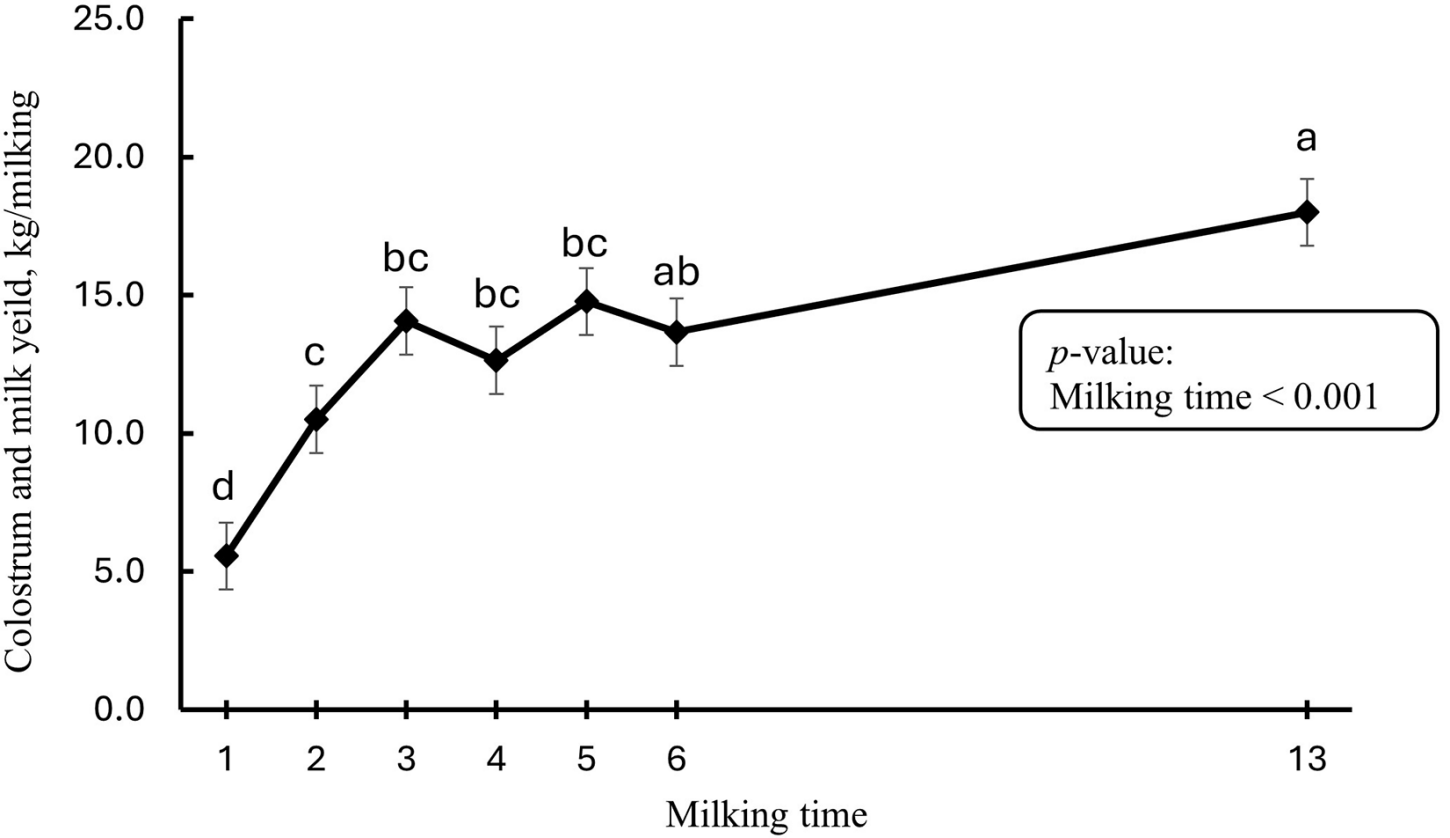
Colostrum and milk yield in the first week post-partum (n = 10). Error bars present SEM. ^a-d^ LS-means with different letters differ according to the adjusted Tukey’s test.

The DMI tended to be higher in the lactating phase (15.9 ± 0.8) compared to the close-up phase (13.5 ± 0.5 kg/d; *p* = 0.086). In addition, daily DMI in the first week post-partum is presented in Fig 1 and it increased from 1 DIM (10.7) to 7 DIM (18.0 kg/d). The colostrum/milk yield was affected by milking time (Fig 2) and it increased form 1 DIM (5.6) to 7 DIM (18.0 kg/milking).

### Vitamin A and E

**Fig 3:**
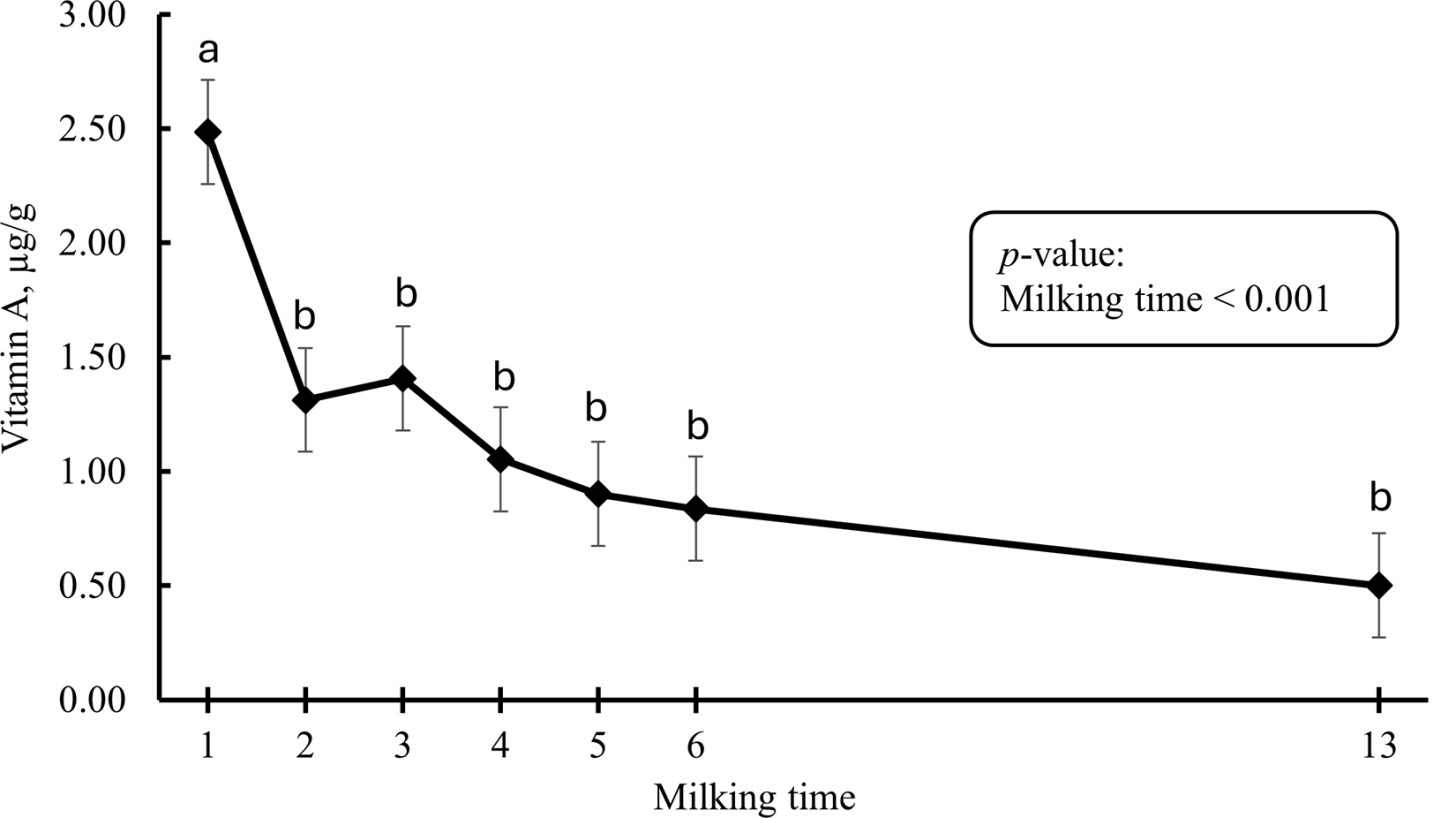
Changes in vitamin A level from colostrum to mature milk (n = 10). Error bars present SEM. ^a-b^ LS-means with different letters differ according to the adjusted Tukey’s test.

**Fig 4:**
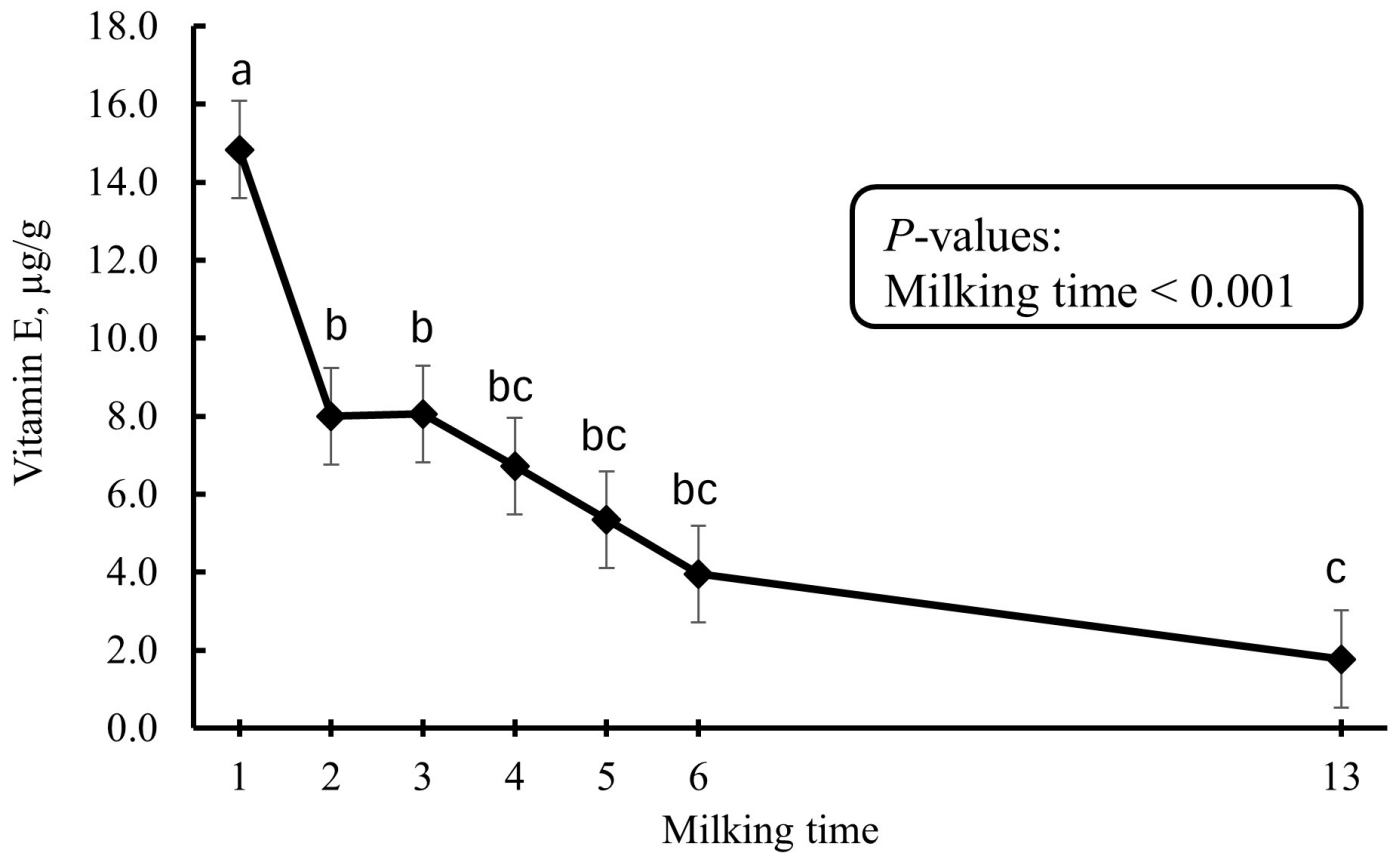
Changes in vitamin E level from colostrum to mature milk. Error bars present SEM. ^a-c^ LS-means with different letters differ according to the adjusted Tukey’s test.

Changes in vitamin A level from colostrum to mature milk are presented in Fig 3. There was an effect of milking time (*p* < 0.001). Colostrum vitamin A was highest in the 1^st^ milking; however, it dropped sharply after the 1^st^ milking and reached a steady level after the 4^th^ milking. Colostrum/milk vitamin E level was affected by milking time (*p* < 0.001; Fig 4). Colostrum vitamin E level continuously dropped after the 1^st^ milking and it reached the lowest level in mature milk.

### Fat and fatty acid profile

Changes in fat percentage, total FA percentage, and FA profile from colostrum to mature milk are presented in Table 2. Milk fat percentage was affected by milking time (*p* = 0.035). Similarly, there was a tendency for milking time (*p* = 0.084) on milk FA percentage. Both fat and total FA percentage dropped from the 1^st^ to 3^rd^ milking, and it increased from 4^th^ milking to mature milk.

Proportion of C4:0 (*p* < 0.001), C6:0 (*p* < 0.001), C8:0 (*p* < 0.001), and C10:0 (*p* = 0.017) FA was affected by milking time and increased from colostrum to mature milk. Proportion of C4:0 FA increased from 14.5 in the colostrum to 24.7 in mature milk. Proportion of C12:0, C14:0, and C16:0 FA was affected by milking time (*p* < 0.001 for all FA) and decreased from colostrum to mature milk. The FA proportion of C18:0 (*p* < 0.001), C18:1n-9 (*p* < 0.001), C18:1n-7 (*p* < 0.001), trans C18:1n-11 (*p* < 0.001), and trans C18:1n-10 (*p* = 0.001) was affected by milking time and increased from colostrum to mature milk.

Proportion of C18:2n-6, C18:3n-6, C20:3n-6, C20:4n-6, and C22:5n-6 FA was affected by milking time (*p* < 0.001 for all FA). Proportion of C18:2n-6 FA decreased from 31.9 in colostrum to 26.1 (g/kg of FA) in mature milk. Similarly, proportion of C20:2n-6, C20:3n-6, C20:4n-6, and C22:5n-6 FA decreased from colostrum to mature milk. However, daily secretion of C20:2n-6, C20:3n-6, C20:4n-6, and C22:5n-6 FA (g/milking; calculated as milk FA proportion × total milk FA × milk yield) was increased due to increased milk yield (results not presented in tables or figures).

Proportion of C18:3n-3 (*p* < 0.001), C20:3n-3 (*p* = 0.019), C20:5n-3 (*p* < 0.001), C22:5n-3 (*p* < 0.001), and C22:6n-3 (*p* < 0.001) FA was affected by milking time. Proportion of C18:3n-3 and C20:3n-3 FA increased from colostrum to mature milk. Proportion of C20:5n-3, C22:5n-3, and C22:6n-3 FA decreased from the colostrum to mature milk. However, daily secretion of C20:5n-3, C22:5n-3, and C22:6n-3 FA (g/milking; calculated as milk FA proportion × total milk FA × milk yield) was increased due to increased milk yield (results not presented in tables or figures).

## Discussion

Colostrum is characterized with a high level of fat-soluble vitamins and fat. The FA composition is different from mature milk and likely reflects the newborn calf requirement. This profile from colostrum to mature milk changes gradually during the transition phase. Therefore, studying this composition is required for appropriate formulating of milk replacers or supplements when maternal colostrum is insufficient.

### Vitamin A and E

The mammalian fetus is identified by low vitamin A status due to low synthesis of retinol-binding protein [13,14] and low transfer of lipid soluble compounds via the placenta from maternal circulation [15], which has a major role in delivering a hydrophobic molecule of retinol to an infant. Similarly, the mammalian fetus is characterized by low vitamin E status due to low synthesis of α-tocopherol transport protein [16], which has a critical role in delivering vitamin E (RRR-α-tocopherol) during pregnancy. The low transfer of plasma lipid to fetus may have an important role for low placenta transfer of vitamin A and E [14]. A steady increased level of vitamin E through the fetal life, mainly through the last third of gestation, seemed to be correlated to the fetal lipid mass [15]. Sinha et al. [17] reported that in human infants, the adipose tissue amount increased from less than 1% to 16% of body weight during the first two trimesters compared to term. A limited and tightly controlled placental transfer of vitamins E and A exposed the newborns to deficiency; thus, it showed the importance of colostrum vitamins A and E during the early stage of life [13]. In support of high colostrum vitamin A level, a noticeable decrease in maternal circulating vitamin A [4,18] and E [18,19] has been reported during late gestation as a consequence of higher mammary uptake of vitamin A and E for colostrum synthesis [20]. According to Chappell et al. [21], the mammary gland compensates for the limited placenta transport by actively enhancing the secretion of vitamins A and E into the colostrum.

In line with our results, it has been reported that vitamin E and A level reduced sharply during the first few days of lactation [3,22] and stabilized after 5 and 3 days post-partum for vitamin A and E, respectively [3]. Rüegg et al. [23] suggested that the reduction in vitamin E level from colostrum to milk can be explained by a progressive increase in the size of fat globules as milk matures. This size change of fat globules may lead to a higher relative percentage of core components of milk fat globules, like triglycerides and cholesterol, which causes a decrease in the relative proportion of the membrane components, such as α-tocopherol and cholesterol [24,25]

### FA profile – Calf physiology and requirement aspect

The plasma of newborn ruminants contains very low levels of unesterified FA, triglycerides, phospholipids, and cholesteryl ester [26]. Additionally earlier study classified cows’ fetuses as being deficient in essential FA compared to non-ruminant species due to a low pool of essential unsaturated FA in the maternal plasma [27]. The primary source of essential FA for the fetus is maternal plasma unesterified FA. However, in cows, it has been well documented that the maternal plasma concentration of unesterified FA is low, and the content of plasma unsaturated FA is negligible [26,28]. Consequently, immediately after birth, newborn calves rely on the unsaturated FA from colostrum and milk, and the low plasma lipid level in newborn ruminants explains the high mammary synthesis of fat and FA during the first week post-partum, providing fat as an essential nutrient at this early stage of life. In support of our results, Noble et al. [28] reported that the concentration of total plasma FA increased from 84 at birth to 230 (mg/100 mL) 24h after birth in lamb, as a consequence of high fat intake provided by colostrum.

Increased proportion of C4:0 FA from colostrum to mature milk can be explained by its beneficial effect on intestinal development, health, and calf growth [29]. Sodium butyrate enhanced the health-associated bacteria in the hindgut of milk-fed calves and maturation of gastrointestinal function [29]. The present results showed a 62% reduction in the FA proportion of C16:0 and a 61% increase in the FA proportion of C18:0 from the colostrum to mature milk. A high pool of C16:0 FA and C16:1n-9 FA in the liver of newborn ruminants may explain the reduction in C16:1n-9 from colostrum to mature milk [28,30]. In addition, the need for C18:0, C20:0, and C22:0 FA for synthesis of phosphatidylethanolamine and phosphatidylcholine [28,31] may explain the increased proportion of C18:0, C20:0, and C22:0 from colostrum to mature milk. It has been well documented that synthesis of phosphatidylethanolamine and phosphatidylcholine at an early stage of life after birth is vital for neurogenesis, cell membranes, and secretions of lipoproteins, bile, and surfactant [32,33].

The proportion of n-6 FA group (C18:2n-6, C18:3n-6, C20:3n-6, C20:4n-6, and C22:5n-6) decreased from colostrum compared to mature milk. In line with our results, O’Callaghan et al. [6] reported that both C18:2n-6 and C20:3n-6 were higher in colostrum compared to mature milk in 5 DIM. The higher proportion of n-6 FA group in colostrum can be explained by the newborn calf’s need for inflammatory mediators, such as eicosanoids when exposed to pathogens [34]. In addition, newborn ruminants are born with a substantial plasma pool of arachidonic acid (C20:4n-6) [30], resulting in a significantly higher plasma C20:4n-6 to C18:2n-6 ratio compared to maternal plasma levels (2.72 in lamb fetus versus 0.21 in ewe). This substantial pool of plasma arachidonic acid in newborn ruminants [30] may explain the observed decrease in the proportion of C18:2n-6 and C20:4n-6 levels from colostrum to mature milk. Despite the increased in proportion of C18:3n-3, the longer chained n-3 (C20:3n-3, C20:5n-3, C22:5n-3, and C22:6n-3) decreased from colostrum to mature milk, which can be justified by activity of Δ5- and Δ6 desaturase enzyme. The high activity of Δ5- and Δ6 desaturase enzyme in human fetal livers [35] shows that newborn infants are capable of synthesizing the long chained PUFA n-6 and n-3 with ≤ C20 FA under sufficient supply C18:2n-6 and C18:3n-3. In addition, the decrease in the proportion of longer chained n-6 and n-3 could be due to the dilution effect of increased milk yield from colostrum to mature milk. In agreement with our findings, the proportions of C20:5n-3, C22:5n-3, and C22:6n-3 decreased from colostrum to mature milk in both primiparous and multiparous cows, despite the relatively stable levels of C18:3n-3 [36].

### FA profile – Mammary lipogenesis aspect

Similar to our results, a variation in fat percentage from colostrum to mature milk has been reported during the first 5 days post-partum [5]. Tsioulpas et al. [5] reported that fat percentage dropped on day 2 post-partum and increased on days 4 and 5. In the present study, the increase in milk fat percentage and FA percentage from 5^th^ milking to mature milk could be explained by decreased milk protein and ash percentage as reported previously [5].

The increase in short chain fatty acids (SCFA; C4:0, C6:0, C8:0, and C10:0) from colostrum to mature milk could be due to a steady increase in DMI, which provides substrates for rumen microbes to produce VFA (mainly acetate and butyrate), serving as precursors for de novo milk FA synthesis. The increased proportion of C18:0 from colostrum to mature milk could be due to increased intake of C18:0 from elevated DMI and increased C18:0 FA originating from rumen biohydrogenation of unsaturated C18 FA [9]. In addition, it has been well established that dairy cows at early lactation face a negative energy balance, which results in fat mobilization from body reserves [37]. Stoop et al. [38] reported that milk C16:0 and C18:0 FA increased in cows with a negative energy balance due to fat mobilization from the body reserve. However, in the present study, we were not able to distinguish between C18:0 originating from the diet, rumen biohydrogenation of unsaturated C18 FA, and fat mobilization from body reserves.

Increased proportion of C18:1n-9 was accompanied by an increase in the proportion of C18:0 from colostrum to mature milk, serving as a substrate for desaturation by Δ9 desaturase enzyme, despite the contribution of C18:1n-9 from DMI [39] and fat mobilization from the body reserve [40]. The steady increased proportion of C18:1n-9 FA from colostrum to mature milk, as the major n-9 FA in the colostrum and milk, indicated the elevation in Δ9 desaturase enzyme activity. However, proportion of C16:1n-7 FA decreased, which can be explained by decreased C16:0 proportion as substrate for desaturation by Δ9 desaturase enzyme. In addition, the increased proportion of C20:0 and C20:1n-9 from colostrum to mature milk, which are synthesized from the elongation of C18:0 and C18:1n-9, reflects the elevation in activity of elongation enzyme.

The increased proportion of rumen biohydrogenation intermediate FA (trans C18:1n-11 and trans C18:1n-10) from colostrum to mature milk could be attributed to the higher FA intake due to increased DMI during the first week post-partum. The increased FA intake supplies more unsaturated FA for microbes involved in rumen biohydrogenation, leading to higher levels of biohydrogenation intermediates. In agreement with our results, dairy cows fed a diet containing 15% DM of crushed sunflower seeds showed an increase of over 300% in rumen biohydrogenation intermediates (trans C18:1n-11 and trans-10 cis-12, and cis-9, trans-11 conjugated linoleic acid) compared to the control group [41].

Even though the proportion of long chained PUFA n-6 (C20:2n-6, C20:3n-6, C20:4n-6, and C22:5n-6) and n-3 (C20:5n-3, C22:5n-3, and C22:6n-3) decreased from colostrum to mature milk, the daily secretion of all the aforementioned long chained PUFA n-6 and n-3 increased from colostrum to mature milk. The long-chained PUFA n-6 and n-3 can be originated from DMI and endogenous synthesis by activity of desaturation (Δ5 and Δ6) and elongation enzymes. However, it seems that the endogenous synthesis has a main role in ruminants due to extensive rumen biohydrogenation of unsaturated FA. This increase in long chained PUFA n-6 and n-3 reflects that, by advancing DIM from 1 to 7, activity of desaturation (Δ5 and Δ6) and elongation enzymes rapidly increase and is upregulated due to increased milk yield.

## Conclusion

The results showed that the first colostrum contained over six times more vitamin E than vitamin A, and colostrum is a rich source of vitamin A and E for newborn calves. There was a considerable reduction in vitamin A and E levels from colostrum to mature milk. Additionally, there were significant changes in the FA profile from colostrum compared to the mature milk, with an increase in the proportion of C18:3n-3 FA and a decrease in the proportion of C18:2n-6 FA from colostrum to mature milk. There was a remarkable increase in C18:0 and C18:1n-9 from the 1^st^ milking to mature milk.
